# Attention, Not Performance, Correlates With Afterdischarge Termination During Cortical Stimulation

**DOI:** 10.3389/fnhum.2020.609188

**Published:** 2021-01-22

**Authors:** Ronald P. Lesser, W. R. S. Webber, Diana L. Miglioretti, Yuko Mizuno-Matsumoto, Ayumi Muramatsu, Yusuke Yamamoto

**Affiliations:** ^1^Departments of Neurology, Johns Hopkins University School of Medicine, Baltimore, MD, United States; ^2^Neurological Surgery, Johns Hopkins University School of Medicine, Baltimore, MD, United States; ^3^Department of Public Health Sciences, School of Medicine, University of California, Davis, Davis, CA, United States; ^4^Kaiser Permanente Washington Health Research Institute, Seattle, WA, United States; ^5^Graduate School of Applied Informatics, University of Hyogo, Kobe, Japan

**Keywords:** cortical stimulation, brain mapping, afterdischarges, attention, cognition

## Abstract

Cortical stimulation has been used for brain mapping for over a century, and a standard assumption is that stimulation interferes with task execution due to local effects at the stimulation site. Stimulation can however produce afterdischarges which interfere with functional localization and can lead to unwanted seizures. We previously showed that (a) cognitive effort can terminate these afterdischarges, (b) when termination thus occurs, there are electrocorticography changes throughout the cortex, not just at sites with afterdischarges or sites thought functionally important for the cognitive task used, and (c) thresholds for afterdischarges and functional responses can change among stimulation trials. We here show that afterdischarge termination can occur prior to overt performance of the cognitive tasks used to terminate them. These findings, taken together, demonstrate that task-related brain changes are not limited to one or a group of functional regions or a specific network, and not limited to the time directly surrounding overt task execution. Discrete locations, networks and times importantly underpin clinical behaviors. However, brain activity that is diffuse in location and extended in time also affect task execution and can affect brain mapping. This may in part reflect fluctuating levels of attention, engagement, or motivation during testing.

## Introduction

Electrical stimulation has been a standard method for mapping brain areas important for motor, sensory, language, and other functions. This is done by delivering trains of electrical pulses to the brain over a period of several seconds. During that time, the patient and testing personnel determine if there are spontaneous changes in sensation, movement, or behavior, or if there is an altered or arrested activity while the patient performs a task (Lesser et al., 1994). However, stimulation can produce afterdischarges (ADs), epileptiform activity occurring after stimulation. These can interfere with mapping and can produce unwanted seizures.

When clinical stimulation produces ADs, additional briefer stimulation pulses can, at times, terminate the epileptiform activity, but this is only successful about half the time (Lesser et al., 1999). When ADs continue, cognitive effort can at times terminate the ADs, with this accompanied by both local (Muldoon et al., 2018) and diffuse (Lesser et al., 2019) electrophysiologic changes in the EEG. Unanswered is what aspect of the cognitive task might better correlate with success in aborting ADs.

Assessing responses to a mental task has at least two components. One is the presentation of the task by testing personnel. Another is the overt task performance by the patient or subject. In this report, we show that afterdischarge termination due to a cognitive task correlates better with presentation than with performance.

## Materials and Methods

We present results from 15 patients who had electrodes implanted for assistance in localizing the regions of onset of their medically intractable seizures. These were primarily subdural electrodes placed on the cortical surface, although some depth electrodes were used to help in recording below the cortical surface, primarily in the region of lesions noted on neuroimaging. Electrode placement, brain stimulation, the cognitive testing used to localize brain functions, and the cognitive activation used in attempts to terminate ADs all were determined by clinical needs alone. Our institutional review board approved our analysis of the recordings and testing.

Details regarding the patients and the testing have been reported previously (Lesser et al., 2019). The following is a summary.

Patients were 12–53 years old at the time of testing, seven males and eight females. Their seizures were not precipitated by mental activation. Testing occurred in the patient's hospital room with the patient supine and with the head of the bed elevated. Patients lay quietly while stimulation parameters at a given site were adjusted (see below and Supplementary Video).

Electrocorticography (ECoG) was recorded from a total of 1,276 platinum electrodes, with 56–109 per patient. Subdural electrodes were discs with 2.3-mm diameter exposed to the cortical surface, and with 10-mm distance between electrode centers. Depth electrodes were 1.1 mm in diameter, with 0.081 cm^2^ surface area, and with 5- or 10-mm spacing (Ad-Tech Medical Instruments Corporation, Oak Creek, Wisconsin 53154, USA.) Electrode positions were verified by direct observation in the operating room, by co-registration of pre-implant 1–1.8 mm coronal slice volumetric MRI studies with post-implant 1-mm axial slice CT scans, and by brain surface renderings based on the co-registered imaging.

Continuous electrocorticography (ECoG) was obtained using Stellate Harmonie Systems (Natus Medical Incorporated, Pleasanton, CA 94566, USA) that could record at 1,000 samples per second per channel, for up to 128 channels. The amplifiers were Schwarzer EEG Amplifiers Model 210033 (Natus Europe GmbH, Robert-Koch-Str. 1, Planegg, Germany). High- and low-pass filters were set to 0.0016 and 300 Hz, respectively. Other details of the recording system are in our previous report (Lesser et al., 2019).

Clinical stimulation for functional localization was always between electrode pairs, using 50 Hz, 0.3 ms duration biphasic square wave pulse pairs, delivered in trains lasting 2–5 s, beginning at 1 mA and gradually increasing to as high as 15 mA, depending on patient or ECoG responses (Lesser et al., 1984, 1994). Patients were observed for motor changes during stimulation and asked if any sensory or cognitive changes occurred. When stimulation intensity was felt optimized, motor, sensory, language, and other testing would occur. ECoG was continuously observed by testing personnel during stimulation testing. Although the patients were in the hospital and had undergone brain surgery for electrode placement, the testing itself was done in a relaxed manner (see Supplementary Video). Patients were not limited with respect to body movement or lack of movement and there were no constraints regarding how quickly they needed to respond to the task, although they tried to answer as quickly as possible.

**Figure 1:**
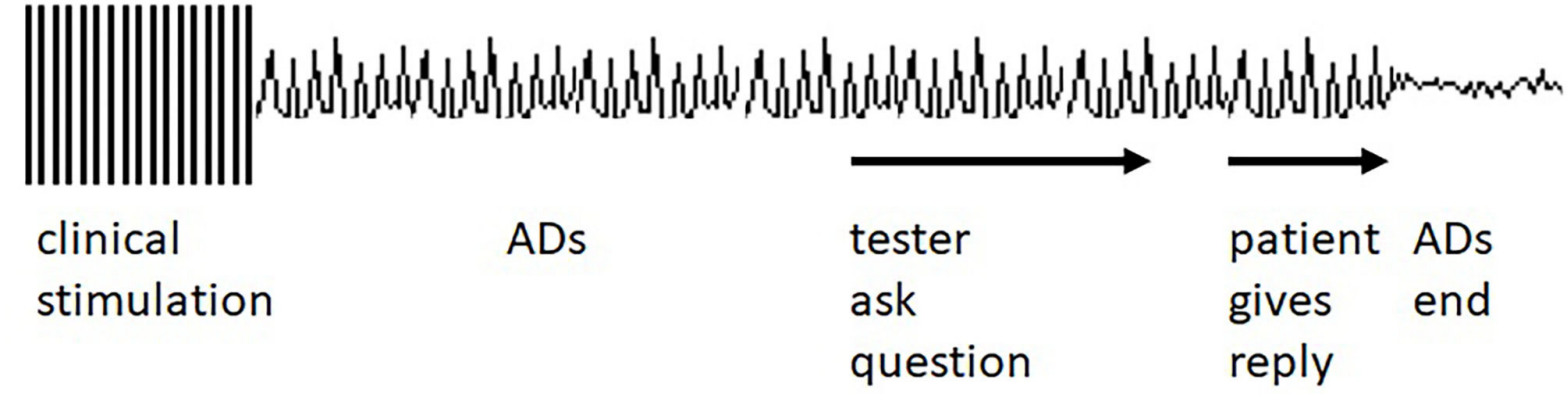
Idealized diagram of the testing setting. Clinical stimulation occurs, and ADs occur as a result. The tester asks the patient a question which the patient answers. The answer is followed by the afterdischarge (AD) ending. See Supplementary Figures 1–3, Supplementary Video for clinical examples. However, ADs often end before the answer is given, as described in the Results section.

When ADs occurred, the testing team would observe the ECoG to see if the ADs stopped spontaneously. If ADs continued, the testing team used brief pulse stimulation (BPS) or a cognitive task, in these cases an arithmetic or spelling task (AST), in attempts to stop the ADs. Instructions regarding the tasks, and the tasks themselves, were presented auditorily. Clinical decisions alone determined whether to attempt AD termination with either BPS or AST. The testing personnel had no specific instructions regarding which to use, or regarding what specific task to use with AST. Patients were likely aware that ADs were occurring because of the reactions of the testing personnel but there were no other warnings that an AST would be presented. Task choices were left to the testing personnel. The tester might use an arithmetic task such as “what is 59 + 8,” “what is 37 + 12,” or “what is 35 – 18?” The patient might also be asked to recite the alphabet backward or to spell a word backward, i.e., “spell horse backward.” Figure 1 diagrams the overall protocol used. The Supplementary Figures 1–3, and the Supplementary Video show clinical examples.

In the previous paper, we found that in 50 of the 116 trials, ADs stopped after a cognitive task was presented. For this report, we studied these 50 trials. We compared (a) the time between initiation of the cognitive task (the Question) and AD termination and (b) the time between initiation of the patient's response (the Answer) and AD termination. We determined the percentage of trials during which AD termination occurred prior to the Answer, calculating the Clopper–Pearson exact binomial confidence interval for the percent.

## Results

**Figure 2:**
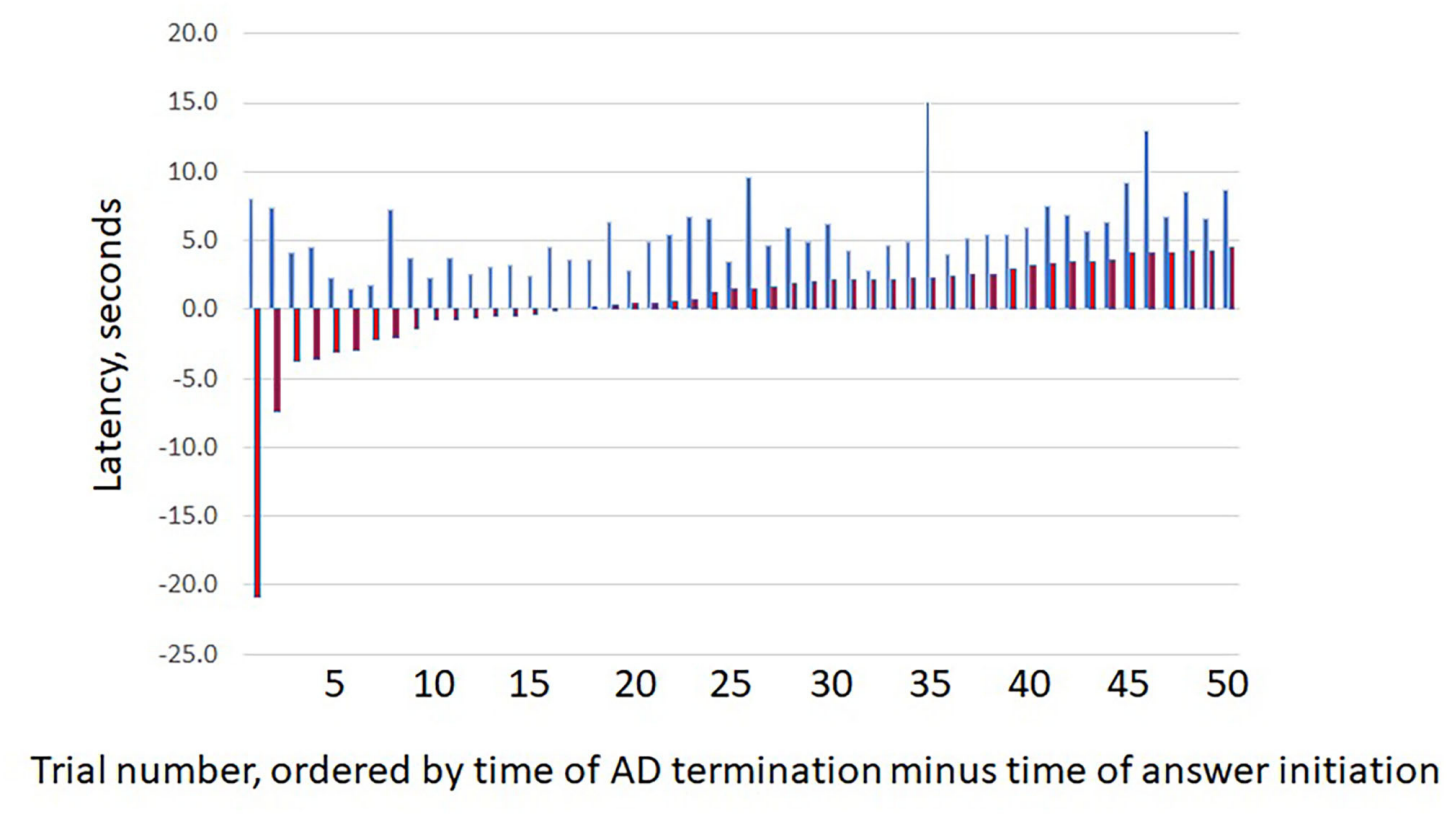
Question and answer latencies. The figure shows the latency between (blue bars) question start and afterdischarge termination (Q) vs. (red bars) answer start and afterdischarge termination (A). For each pair, the first bar (blue) indicates the value for Q and the second bar (red) the value for (A). As would be expected, ADs always end after the question was asked. However, ADs often ended before an answer was given, as shown by the negative latencies for the first red bars. X axis shows results for the 50 trials, with pairs sorted by increasing latency for (A). Y values are in seconds.

We measured the time differences between when the question or answer was initiated and when the ADs stopped. As would be expected, given the protocol, ADs always ended after a question was asked. However, ADs often stopped before the answer was given (Figure 2). This occurred for 16/50 trials (32%, 95% CI = 19.5%, 46.7%). Regardless of when answers began, there was no clear temporal relationship between times of answer onset and of AD termination.

In our previous paper (Lesser et al., 2019), we reported functional magnetic resonance imaging (fMRI) results on six patients and nine controls, using ASTs similar to those used during stimulation testing. In summary, the tasks activated multiple regions, including regions thought important for mental effort. They, however, were not necessarily sites where stimulation had resulted in ADs, and the ADs were not necessarily in regions important for the ASTs (also see Supplementary Material).

Therefore, our results, overall, indicate that AD termination due to an AST can occur prior to overt performance of the task and can occur regardless of the location of the ADs.

## Discussion

It is well-known that motor activities are accompanied by electrophysiological changes such as the bereitschaftspotential, the premotor potential, and neuronal firing in both human (Shibasaki et al., 1980a,b; Thomas et al., 2019) and non-human (Dorris et al., 2000; Boussaoud, 2001) studies. Similar changes accompany the performance of cognitive tasks (Haller et al., 2018). These potentials begin before, and continue after, the task is performed and appears to be temporally related to the timing of task performance. If AD terminations either occurred just before the onset of an answer, perhaps similar to the timing of a premotor potential, or occurred with a clear temporal linkage to the answer, this would favor the possibility that termination was related to task performance. However, in our patients, we found that AD termination often occurred before the patient performed the task and without a tight temporal relationship to task performance. This favors the conclusion that AD termination was due to the task situation, but not to the answer itself. Auditory modalities were used both for the questions and answers with these patients. Future studies might compare auditory versus visual tasks, but we think it unlikely that task modality itself was responsible for our findings: The widespread ECoG coherence that we previously have reported with ASTs (Lesser et al., 2019) was not restricted to regions important for modulating either audition or the AST modalities used. Thresholds for afterdischarges and for clinical changes in response to brain stimulation are in any case variable (Lesser et al., 1984, 2008; Pouratian et al., 2004).

Protocols for testing brain activity are often constrained by defined stimulus onset and response times. In contrast, with these patients, the AST was given in a more relaxed, perhaps more naturalistic, setting. The patients likely knew to respond quickly but had no constraints other than this. It is possible that these differences explain why AD termination could precede task performance. We suggest that AST termination was associated with internal processes regulating awareness and that were themselves widespread, not stimulus locked, not modality or task specific, and not characterized by a distinct temporal structure (Engel and Singer, 2001). Our findings suggest an extension of this idea and emphasize that in studying brain activity related to performance of a task, one must look beyond overt task performance: the overall level of attention to, engagement regarding, and motivation for completing the task also should be considered (Baddeley, 2003). Those factors are less likely to be precisely related to the time of task performance and more likely to reflect the overall conditions under which the activity is occurring. All of us vary in our level of attention, engagement, and motivation from moment to moment in our daily lives, whether we are researchers, subjects, or patients.

## Data Availability Statement

The raw data supporting the conclusions of this article will be made available by the authors, without undue reservation.

## Ethics Statement

The studies involving human participants were reviewed and approved by the Johns Hopkins Medicine Institutional Review Board. Note that the subject of this report was considered exempt as it reported retrospective review of clinical investigations. Where applicable for the previous fMRI studies referenced in this report, written consents to participate in fMRI investigations were obtained.

## Author Contributions

RL conceived the AST paradigms for the clinical evaluations. Members of the YM–M Lab suggested analysis of question and answer times. RL and WW extracted the question, answer, and AD termination times from the data set and analyzed the results. DM performed statistical analyses of the results. RL wrote the paper, together with WW and DM. All co-authors reviewed the manuscript.

## Conflict of Interest

RL or his wife has stock in the following companies which sell health care products: 3M, Abbott Labs, Abbvie, Apple, Avanos, Celgene, Express Scripts, Johnson and Johnson, Merck & Company, Pfizer. These have been disclosed to and approved by the Johns Hopkins University in accordance with its conflict of interest policies. The remaining authors declare that the research was conducted in the absence of any commercial or financial relationships that could be construed as a potential conflict of interest.

## Supplementary Material

Supplementary Video Testing results on patient S1. Movie is from Lesser et al. (2019). It is included with permission and published by Elsevier B. V. and is © International Federation of Clinical Neurophysiology 2019.

Supplementary Figures 1–3 Explanation of testing paradigm in three patients. Figures are from Lesser et al. (2019). They are included with permission and published by Elsevier B. V. and is © International Federation of Clinical Neurophysiology 2019.

## Abbreviations

AD: afterdischarge
ADs: afterdischarges
AST: arithmetic or spelling task
ECoG: electrocorticography
fMRI: functional magnetic resonance imaging.
