# Supplementary material for: Attention, Not Performance, Correlates With Afterdischarge Termination During Cortical Stimulation
Source: Front Hum Neurosci. 2021 Jan 22;14:609188. doi: 10.3389/fnhum.2020.609188 (PMC7862320; doi:10.3389/fnhum.2020.609188)
Supplement: Supplementary Figures 1–3 — Explanation of testing paradigm in three patients. Figures are from Lesser et al. (2019). They are included with permission and published by Elsevier B. V. and is © International Federation of Clinical Neurophysiology 2019. [file Data_Sheet_1.pdf]

Patient Information																			Arithmetic Results		Spelling Results		
**patient**	**gender**	**age onset**	**age surgery**	**seizure frequency**	**sz type**	**Wada language**	**Wada memory**	**handed**	**patient**	**AC Rx**	**surgery**	**lobe resected**	**imaging prior to this evaluation**	**FU Engel yrs**	**patient**	**electrodes implanted**	**electrodes monitored**	**combinations analyzed**	**M1**	**M0**	**S1**	**S0**	**SUM**
**S1**	f	21y	41y	2/m	FA FI	L	B	R	**S1**	OXC	L F res	F	L mes T ↑ T2, L mes T↓ size	IA / 12	**S1**	91	86	3655	18 / 7	14 / 3	2 / 2	2 / 2	36 / 14
**S2**	m	16y	30y	2/d	FA FB			R	**S2**	LTG	R P les	P	R postcentral sulcus cortical dysplasia	IA / 7	**S2**	81	78	3003	5/4	5 / 3			10 / 7
**S3**	f	3m	20y	1-sev/m	FA			R	**S3**	LTG ZNS	R F les	R	R F focal dysgenesis	IA / 7	**S3**	88	81	3240	5 / 5	3 / 3			8 / 8
**S4**	m	10y	16y	3-4/d	FA FI			R	**S4**	VPA	L T les	L	L T calcified mass	?	**S4**	61	56	1540	1 / 0	3 / 0	2 / 0	4 / 0	10 / 0
**S5**	m	8 h	12 y	sev/m	FA FI FB			L>R	**S5**	VPA LOR	rt post F les	F	R precentral encephalomalacia	IIA / 9	**S5**	81	75	2775	2 / 0				2 / 0
**S6**	m	46y	50y	1/w	FI FB	L	B	L>R	**S6**	CBZ	L B res, leave H	T	L T ↑ FLAIR T2	IA / 9	**S6**	97	91	4095	1 / 0				1 / 0
**S7**	f	10y	27y	up to 60/d	FA FB			R	**S7**	LTG	Ant RF re-res	F	ictal SPECT – RF,RI uptake. PET 20y min ↓ RT, 21y- ↑ RF, RI	IIIA / 7	**S7**	110	108	5778	3 / 2	2 / 2			5 / 4
**S8**	f	7y	53y	2-3/w	FA FI FB	L	L	R	**S8**	LEV CBZ	R T re-res	T	R H ↑ T2, ↓ size	IIIA / 10	**S8**	62	59	1711	1 / 0	3 / 0		1 / 0	5 / 0
**S9**	f	12y	32y	3-5/w	FI FB	L	L	R	**S9**	PHN	extend RT res	T	R periH cyst	IA / 6	**S9**	83	79	3081	1 / 0				1 / 0
**S10**	m	"child-hood"	48y	1/m	FA FB			R	**S10**	LTG	rt F	F	R MCA atrophy	IC / 7	**S10**	113	109	5886		9 / 0		1 / 0	10 / 0
**S11**	m	32y	41y	daily	FI	L	B, L>R	R	**S11**	VPA CBZ	L T Lob sparing H	T	L H ↑ FLAIR. LT PET ↓ FDG, multiple aneurysms	IC / 9	**S11**	81	77	2926	1 / 0				1 / 0
**S12**	f	47y	50y	up to 10/d	FA FI	L	R	R	**S12**	OXC PGB	L T Lob sparing base & H	T	L FT operculum/insula oligodendroglioma	IB / 8	**S12**	108	104	5356	2 / 1	1 / 1			3 / 2
**S13**	f	13y	20y	2-4/w	FI			R	**S13**	LEV CBZ	R T re-res	T	↑ T2, prev RT partial lobectomy	IA / 6	**S13**	86	80	3160		3 / 0			3 / 0
**S14**	m	8y	14y	qod	FI			R	**S14**	OXC RUF LCM VNS	deferred. Subsequent les	O	L mesial occipital closed lip schizencephaly; R thalamus ↑ T2FLAIR	IA / 5	**S14**	96	88	3828		3 / 0	6 / 0	10 / 2	19 / 2
**S15**	f	15y	29y	sev/m	FI	L	B	R	**S15**	LCM LEV	no surgery - risk to language	--	MRI - normal	no surgery	**S15**	115	105	5460		2 / 0			2 / 0
summary	7m 8f	0 - 46 y	12-53 y													1353	1276	55494	40 / 19	48 / 12	10 / 2	18 / 4	116 / 37

ABBREVIATIONS

patient S1-15 indicates patient # 1-15

gender f=female, m=male

age onset, age surgery, seizure frequency h=hours, d=day, w=week, m= month(s), y=years, sev=several, qod=every other day

Sz type (seizure type) FA=focal aware FI=focal impaired awareness FB=focal to bilateral tonic-clonic, using the terminology suggested by the International League Against Epilepsy Commission for Classification and Terminology*

*Fisher RS, Cross JH, French JA, Higurashi N, Hirsch E, Jansen FE, et al. Operational classification of seizure types by the International League Against Epilepsy: Position Paper of the ILAE Commission for Classification and Terminology. Epilepsia 2017; 58(4): 522-30.

Wada (if performed), handed handed=left/right handed L=left dominant, R=right dominant, B=bilateral representation, L>R = left predominance

AC RX anticonvulant treatment at time of surgery

CBZ=carbamazepine, LCM=lacosamide, LEV=levetiracetam, LOR=lorazepam, LTG=lamotrigine, OXC=oxcarbazepine, PGB=pregabalin, PHN=phenytoin, VNS=vagal nerve stimulator VPA=divalproex, ZNS=zonisamide

Surgery lobe resected Imaging prior to this evaluation

Surgery=surgery performed consequent to this evaluation.

A = anterior P= posterior L= left R= right B= temporal base F = frontal I=insula mes=mesial P=parietal T=temporal H= hippocampus MCA= middle cerebral artery distribution

Les= lesionectomy Lob=lobectomy Res=resection Re-res = re-resection prev=previous.

↑ ↓ = increased or decreased signal on imaging study. T2=T2 weighted magnetic resonance imaging study, FDG=fluorodeoxyglucose PET study, FLAIR=Fluid-attenuated inversion recovery magnetic resonance imaging study

MRI=magnetic resonance imaging PET=positron emission tomography SPECT=single-photon emission computerized tomography if not specifically labeled, imaging findings are those from MRI

FU Engel* yrs Outcome on follow-up using Engel criteria / years since surgery at follow-up. ? = lost to follow up

*Engel Jr J. Outcome with Respect to Epileptic Seizures. In: Engel Jr J, editor. Surgical Treatments of the Epilepsies. New York: Raven Press; 1987. p. 553-71.

electrodes implanted = number of implanted electrodes in this patient; **electrodes monitored** = electrodes actually monitored. Others were ground electrodes or malfunctioned

combinations analyzed - total number of pair combinations analyzed. See section 2.6 of report for further details.

M1, M0, S1, S0 = Math (M) or Spelling (S) problem followed (M1, S1) or not followed (M0, S0) by termination of ADs.

X / Y = Total trials / trials during which there was no brief pulse stimulation (BPS) prior to the Math (M) or Spelling (S) problem

Supplemental Table - information regarding the 15 patients

(modified from Lesser et al. *Clin Neurophysiol*.130:2169-2181, with permission and published by Elsevier B.V. and © International Federation of Clinical Neurophysiology 2019.)

Explanation of part 1 of Movie, patient S1

Spontaneous speech is being tested, and therefore the patient is speaking during the initial part of the video. Content of spontaneous speech was chosen by the patient at the time of testing.

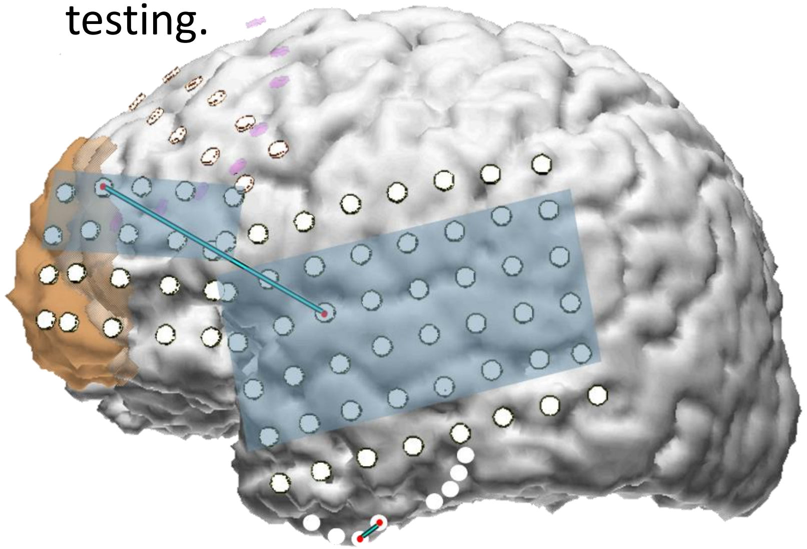

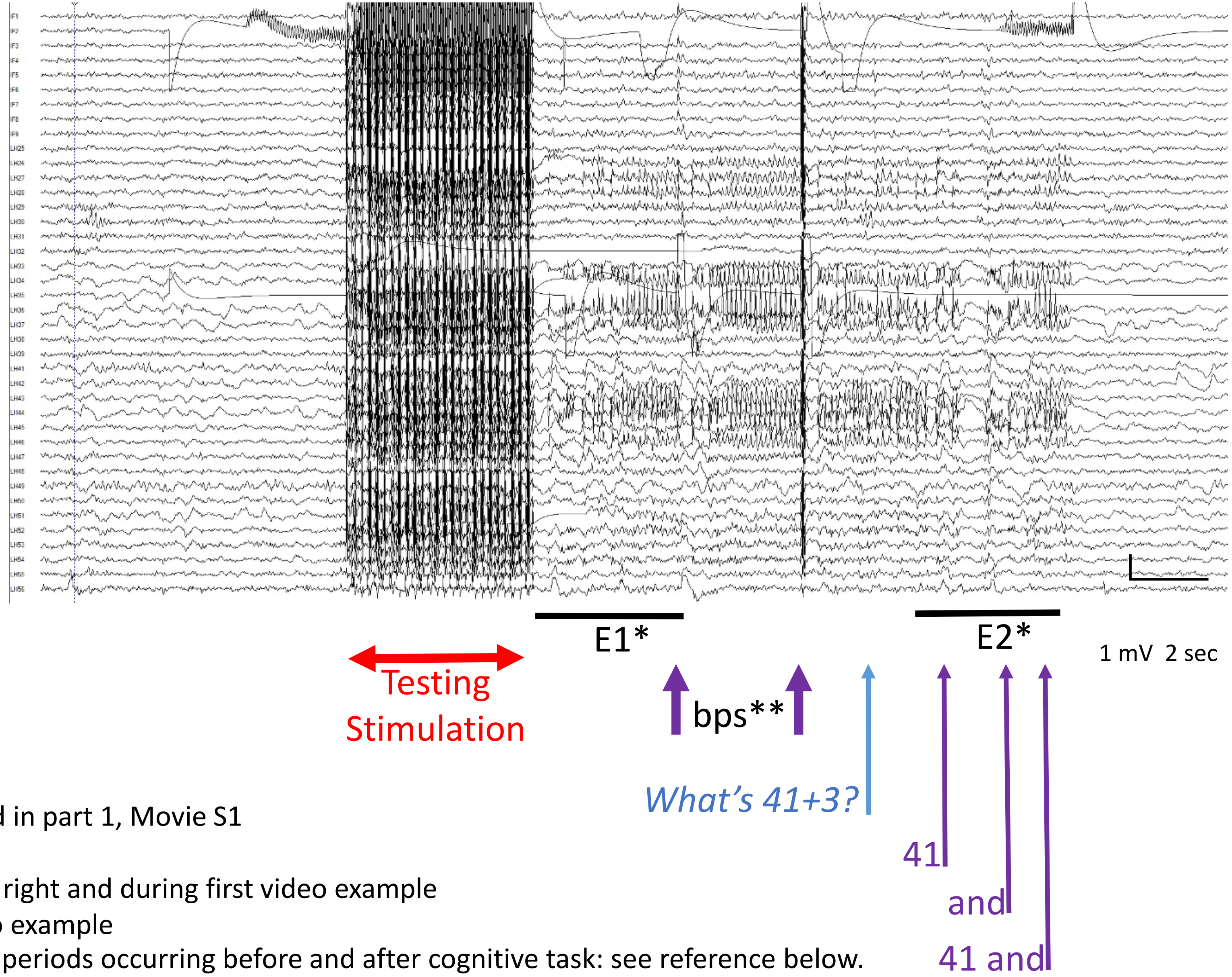

Electrodes shown in recording, above right and in part 1, Movie S1

Stimulated electrode pairs:

Top pair: stimulated during ECoG shown top right and during first video example

Bottom pair: stimulated during second video example

* E1 and E2 are analyzed electrocorticography periods occurring before and after cognitive task: see reference below.

** brief pulse stimulation, see Methods

Fig 1 (modified from Lesser et al. *Clin Neurophysiol*.130:2169-2181, with permission and published by Elsevier B.V. and © International Federation of Clinical Neurophysiology 2019.)

Explanation of part 2 of Movie, patient S1

No testing modality: stimulation intensity is being gradually increased

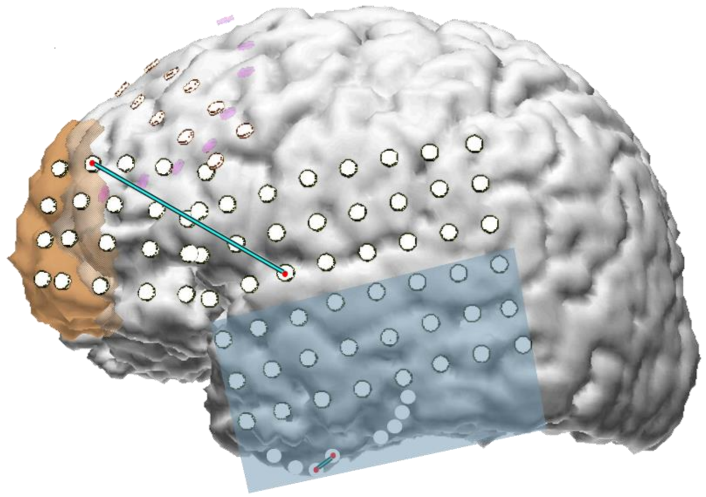

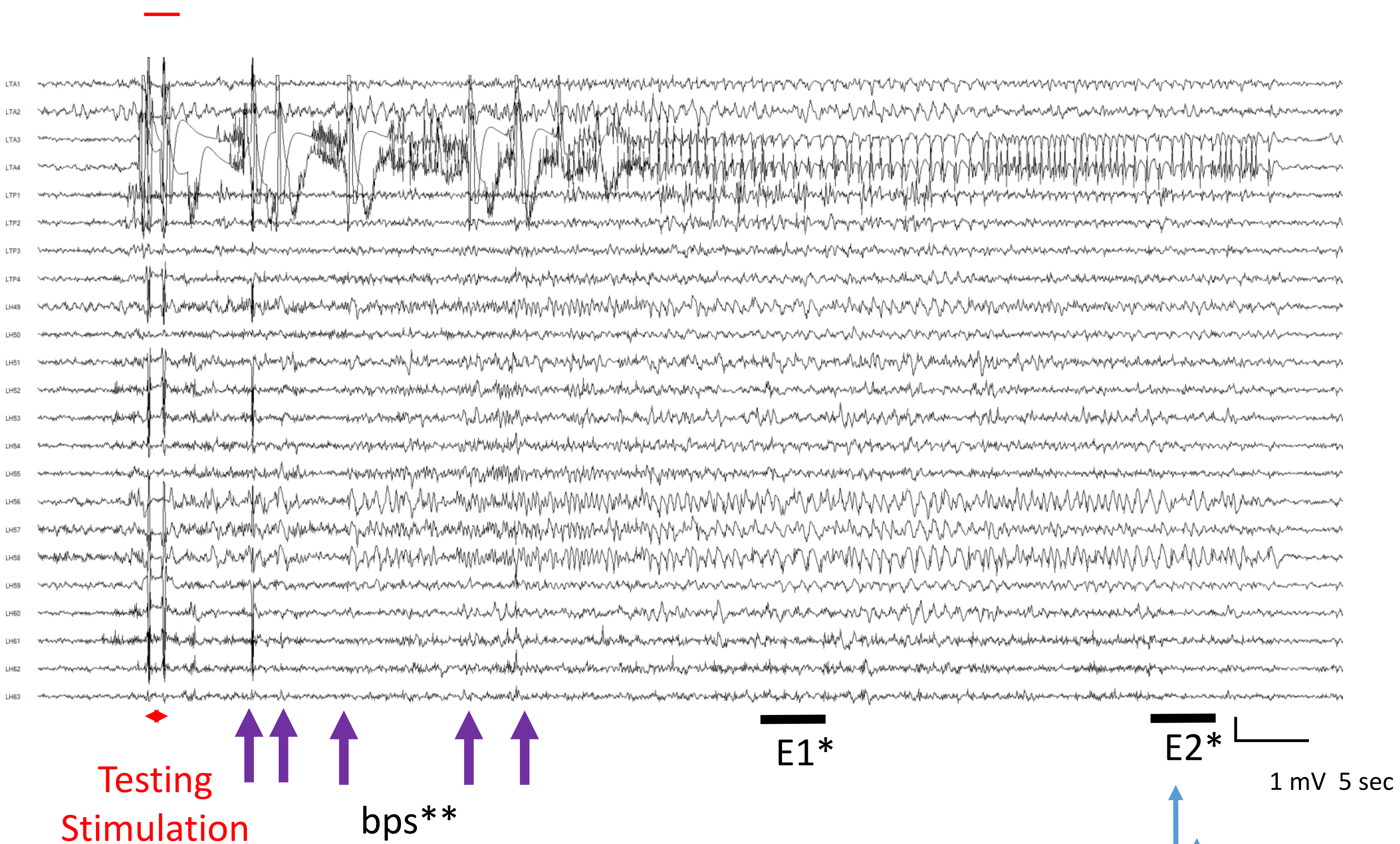

Electrodes shown in recording, above right and in part 2, Movie S1

Stimulated electrode pairs

Top pair: stimulated during ECoG shown top right and during first video example

Bottom pair: stimulated during second video example

* E1 and E2 are analyzed electrocorticography periods occurring before and after cognitive task: see reference below.

** brief pulse stimulation, see Methods

Call patient's name

What's 34+5?

39

Minus 12

Fig 2 (modified from Lesser et al. *Clin Neurophysiol*.130:2169-2181, with permission and published by Elsevier B.V. and © International Federation of Clinical Neurophysiology 2019.)

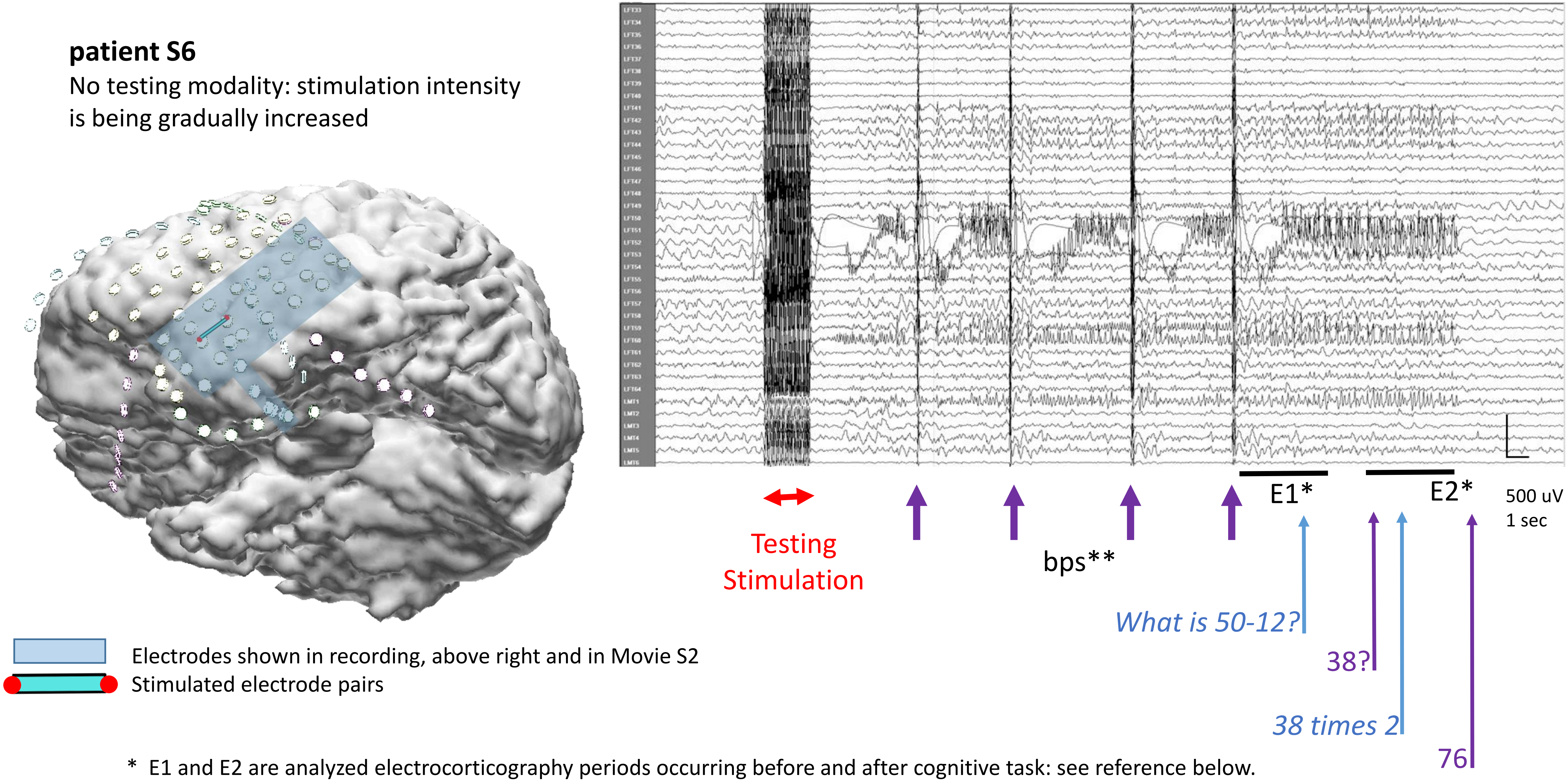

* E1 and E2 are analyzed electrocorticography periods occurring before and after cognitive task: see reference below.

** brief pulse stimulation, see Methods

Fig 3 (modified from Lesser et al. *Clin Neurophysiol*.130:2169-2181, with permission and published by Elsevier B.V. and © International Federation of Clinical Neurophysiology 2019.)
